# Biomaterials and Advanced Biofabrication Techniques in hiPSCs Based Neuromyopathic Disease Modeling

**DOI:** 10.3389/fbioe.2019.00373

**Published:** 2019-11-29

**Authors:** Jing Sun, Xun Ma, Ho Ting Chu, Bo Feng, Rocky S. Tuan, Yangzi Jiang

**Affiliations:** ^1^Faculty of Medicine, School of Biomedical Sciences, Institute for Tissue Engineering and Regenerative Medicine, The Chinese University of Hong Kong, Hong Kong, China; ^2^Faculty of Medicine, School of Biomedical Sciences, The Chinese University of Hong Kong, Hong Kong, China; ^3^Key Laboratory for Regenerative Medicine, Ministry of Education, Faculty of Medicine, School of Biomedical Sciences, The Chinese University of Hong Kong, Hong Kong, China

**Keywords:** biomaterial, hiPSC, biofabrication, disease modeling, Duchenne Muscular Dystrophy, Congenital heart diseases, Alzheimer's disease

## Abstract

Induced pluripotent stem cells (iPSCs) are reprogrammed somatic cells by defined factors, and have great application potentials in tissue regeneration and disease modeling. Biomaterials have been widely used in stem cell-based studies, and are involved in human iPSCs based studies, but they were not enough emphasized and recognized. Biomaterials can mimic the extracellular matrix and microenvironment, and act as powerful tools to promote iPSCs proliferation, differentiation, maturation, and migration. Many classic and advanced biofabrication technologies, such as cell-sheet approach, electrospinning, and 3D-bioprinting, are used to provide physical cues in macro-/micro-patterning, and in combination with other biological factors to support iPSCs applications. In this review, we highlight the biomaterials and fabrication technologies used in human iPSC-based tissue engineering to model neuromyopathic diseases, particularly those with genetic mutations, such as Duchenne Muscular Dystrophy (DMD), Congenital Heart Diseases (CHD) and Alzheimer's disease (AD).

## Introduction

Induced pluripotent stem cells (iPSCs) are pluripotent stem cells generated from somatic cells that maintain many of the features of embryonic stem cells (ESCs) such as pluripotency and self-renewal ability. iPSCs were first generated by Shinya Yamanaka from mouse and human fibroblasts using four factors: octamer-binding transcription factor 3/4 (Oct3/4), sex determining region Y-box 2 (Sox2), c-Myc, and kruppel-like factor 4 (Klf4) (Takahashi and Yamanaka, 2006; Takahashi et al., 2007). Later on, many different substitutes for these factors were discovered and used, e.g., Estrogen-related receptor b(Esrrb), Klf2 for Klf4; Sox17EK for Sox2; Nr5a2 and transforming growth factor beta (TGF-β) inhibitor SB43152 for Oct3/4, and L-Myc for c-Myc (Feng et al., 2009; Heng et al., 2010; Xiao et al., 2016).

iPSCs can be generated from various somatic cell types that are easily obtained (Gnecchi et al., 2017), and give rise to many terminal differentiated cell types that have been used in tissue engineering for studying disease and developing treatments (Lin et al., 2017). One advanced application of iPSCs is for precision or personalized medicine, since the iPSC technology enable us to generate unlimited amount of cells from a specific patient for drug efficacy prediction or tissue regeneration (Gnecchi et al., 2017), and this can overcome the ethical issues that human ESCs usually meet (Pen and Jensen, 2017).

After the hiPSC cell-lines were successfully established and maintained, there are step-wised differentiation processes to get certain mature tissue cells. The first step is to differentiate iPSCs into three primary germ layers, the ectoderm, mesoderm, and endoderm. The differentiation protocol of these steps has been well-established, and different groups of growth factors and bioactive molecules are involved. For example, the ectodermal bone morphogenetic protein 4 (BMP4) and ɤ-secretase inhibitor (N-[(3,5-Difluorophenyl)acetyl]-L-alanyl-2-phenyl]glycine-1,1-dimethylethyl ester) (DAPT) can induce iPSCs differentiate into the surface ectoderm (SE) after 2 days of culture (Qu et al., 2016). After the iPSCs derived cells differentiated into 3 germ layers, regulating the TGF-β, WNT, or fibroblast growth factor (FGF) signaling pathways that mimic each stage of development can further push iPSCs into different sequential lineages. During studying the biology of iPSCs, biomaterials also served as a strong supportive factor that improve the differentiation, proliferation, and application of iPSCs, and are gradually getting attentions (Guo et al., 2017; Khan and Tanaka, 2017).

Biomaterials have been developed to provide biophysical (e.g., stiffness and topography) and biochemical (e.g., growth factors and signaling pathway) cues to cells, which mimic the stem cell niches and microenvironment *in vitro*, and have been well-used in stem cell related studies and applications. For example, higher efficacy and accuracy can be achieved in stem cell differentiation when biomaterials involved (Kumari et al., 2010), and biomaterials can provide homing signals for stem cell migration and localization (Martino et al., 2012). In addition, biomaterials can build scaffolds to support and guide the cell behavior during forming three-dimensional (3D) tissue and organs, which are indispensable elements in tissue engineering, drug screening and disease modeling.

The neuromyopathic diseases are the most prevalent diseases in the world, including cardiomyopathy, motor neuron disease, peripheral nervous system disease, and associated muscular disease. Some of the diseases are due to genetic mutations, for example, Alzheimer's disease with *PSEN2*^*N*141*I*^ gene mutation affected 29.8 million people worldwide in 2015 (GBD 2015 Disease Injury Incidence Prevalence Collaborators, 2016). Congenital heart diseases (CHD) with *DAND5* gene mutation is the most common birth defect affecting between 4 and 75 per 1,000 at birth and resulting in 303,300 deaths in 2015 (GBD 2015 Disease Injury Incidence Prevalence Collaborators, 2016), which have great potential to be modeled by patient derived iPSCs. One more example is the Duchenne Muscular Dystrophy (DMD), the most common type of muscular dystrophy, has mutation in dystrophin gene affecting about one in 5,000 males at birth (Moat et al., 2013). The accordingly genetic mutant animals are used to elucidate disease mechanisms, such as the *mdx* mouse, which has a point mutation in its DMD gene, that produces non-functional dystrophin protein in muscle, thus generate the DMD disease in mouse. However, the *mdx* mouse model only show a non-consistent disease progress and exhibit mildly dystrophic (Spencer and Tidball, 1996; Grounds and Torrisi, 2004), and do not completely recapitulate the phenotype of human DMD disease due to the genetically distinction between animals and human. Using patient derived iPSCs and the tissue engineering technique to build DMD models for studying disease and therapies (Choi et al., 2016), and can overcome the limitation of animal models (Park et al., 2008).

**Figure 1:**
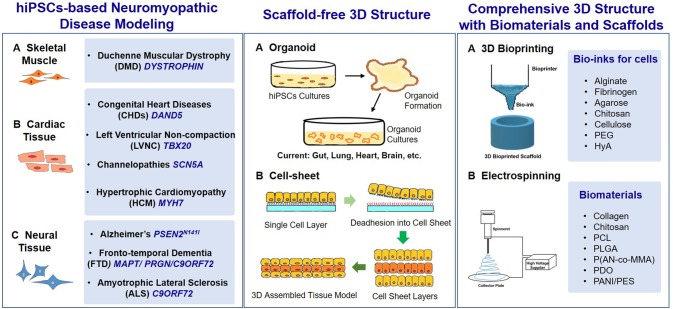
Key elements in hiPSC-based neuromyopathic disease modeling. **(Left)** The cells used in disease modeling can be derived from patient-specific iPSCs, which carry the genetic mutations in (A) skeletal muscle, (B) cardiac tissue, and (C) neural tissue, and cause the neuromyopathic diseases. The gene names are in deep blue, italic, and capitalized. **(Middle)** (A) Organoid and (B) cell-sheet technologies are the most common examples of scaffold-free hiPSCs based tissue engineering. **(Right)** Biomaterials and scaffold are used to achieve comprehensive 3D structures with advanced biofabrication processes, (A) 3D bioprinting and (B) electrospinning are widely used in combination with many biomaterials as bio-inks and scaffolds. PDO, polydioxanone; P(AN-co-MMA), poly(acrylonitrile-co-methyl methacrylate); PANI/PES, polyaniline (PANI)/poly(ether sulphone) (PES).

3D structure of iPSCs and the iPSC-derivations cultures is requested in the terminal differentiation steps to construct many tissues and organs that simulate the native conditions. Biomaterials and related biofabrication techniques have been used in hiPSCs fate decision and application, but they were not enough emphasized and recognized (Yildirimer et al., 2019). For instance, cell-sheet self-assembly technique was used in hiPSCs based clinical study of treating exudative age-related macular degeneration (Mandai et al., 2017), and the electrospinning, computing aided design/rapid prototyping, 3D bioprinting (Wheelton et al., 2016) are being investigated. In this review, we highlight the involvement of biomaterials and the biofabrication techniques in hiPSCs-based tissue engineering, particularly in hiPSCs-based *in vitro* modeling of neuromyopathic diseases (Figure 1).

## Biomaterials for iPSCs Application

The iPSC supportive biomaterials should be biocompatible, biodegradable, and have enough mechanical strength. In this section, the classification, composition, physical, and chemical cues of suitable biomaterials are discussed.

### Classification of Biomaterials in iPSCs and Tissue Engineering

The common biomaterial types in stem cell and tissue engineering are inorganic materials, natural polymers, and synthetic polymers. The inorganic materials, such as metals and ceramics, have been widely applied as substitutes for broken bone or teeth, but these hard tissue specific characteristics also make inorganic materials rarely used in other applications. Meanwhile, polymer biomaterials, either natural or synthetic fit many application scenarios with stem cells involved, and have the potential to be directly adopted in iPSC applications.

#### Natural Derived Materials

Natural derived materials are largely similar to the cellular microenvironment, or even are directly taken from the extracellular matrix (ECM), which indicate the great biocompatibility with hiPSCs.

The mostly used natural polymers as scaffold and cell vehicles are polysaccharides. The polysaccharides are either from plants (e.g., alginate, agarose, and cellulose) or from animals (e.g., chitosan and chitin). The aqueous solutions of these polysaccharides can undergo a sol-gel transition upon reversible effect of external stimuli, such as temperature (agarose) and ionic strength (alginate and chitosan), forming polysaccharide-based hydrogel, which have good biocompatibility for cell survival and high porosity for cell ingrowth and effective mass transport. When processed at physiology-like condition, the hydrogels are capable to encapsulate cells. The mechanical properties and average pore size of polysaccharide-based hydrogel are dose- and structure-dependent (Aymard et al., 2001; Drury et al., 2004). Agarose and cellulose are slow/non-degradable *in vivo* and *in vitro*, and are used for long-term cell culture support. Meanwhile, alginate, chitosan and chitin are biodegradable, which are commonly used in drug delivery and making scaffolds for tissue reconstruction, e.g., for blood vessel, nerves and bone. In order to build a complex tissue, many different polymers are employed during tissue engineering applications. For instance, the combination of alginate, carboxymethyl-chitosan and agarose were used as cell-laden bio-ink to print iPSC-encapsulated construct for neuron reconstruction, and this construct can maintain the proliferation and pluripotency of iPSCs, and induce neuron differentiation and tissue reconstruction (Gu et al., 2017).

A typical natural derived ECM component is type I collagen, the most abundant ECM proteins in native tissue, and it is the first gel used in 3D tissue engineering (Vandenburgh et al., 1988; Kjaer, 2004). The derivant of collagen, gelatin, which can reduce the adverse effect of immunogenic problems of type I collagen (Schwick and Heide, 1969), then became popular in the *in vivo* applications (Tondera et al., 2016). One application example in iPSCs is that the fibroblast reprogramming efficacy was increased when seeded on type I collagen scaffold compares to the conventional 2D culture method (Gu et al., 2016).

Other ECMs, such as Matrigel or related proteins (e.g., laminin and fibrin), are also commonly applied in supporting iPSCs formation (Feaster et al., 2015), and help iPSCs derived cells forming 3D structures during differentiation and tissue reconstruction (Kong et al., 2018; Maffioletti et al., 2018). For example, thick mattress of undiluted Matrigel enables rapid generation and enhanced maturation of rod-shaped hiPSC-derived cardiomyocytes with aligned myofilaments and robust contractile responses. This Matrigel mattress-based cell culturing allows quantification of contractile performance at the single cell level, which should be valuable to disease modeling, drug discovery and preclinical cardiotoxicity test (Feaster et al., 2015). Using cell derived matrix such as fibroblast derived ECM to culture hiPSCs can mediate cell plasticity (Kim et al., 2018). Furthermore, PuraMatrix, a new commercial synthetic matrix, is a natural polymer mimic 16 amino acid synthetic peptide that can self-assemble into nanofibers (~10 nm) in response to monovalent cations to mimic the *in vivo* soft hydrolgel based ECM for neural lineage differentiation (Zhang et al., 2014). However, low mechanical strength, potential impurities and inconsistence among the manufacturing batches limit the application of some of the ECM derived materials.

#### Synthetic Polymers

Synthetic origin polymers had been utilized in iPSCs based tissue engineering too. The well-established synthetic polymer types are poly(ε-caprolactone) (PCL), poly(3-hydroxybutyrate) (PHB), poly(lactic acid) (PLA), poly(L-lactic acid) (PLLA), poly (glycolic acid) (PGA), and their copolymers like poly(lactic-co-glycolide) (PLGA). Among these, PLGA and PCL, both have been approved by American Food and Drug Administration (FDA) for clinical application, are most frequently used. Both of PLGA and PCL can either form different shapes of biocompatible scaffolds, or encapsulate and deliver drug/bioactive molecules based on their controllable biodegradability. PCL is more stable in quality and more cost-effective than PLGA, while PLGA is easier in processing. Another elastic tissue substitute synthetic polymer is polydimethylsiloxane (PDMS), which has flexible mechanical strength, excellent biocompatibility, non-degradability, low cell toxicity, and negligible immune reaction, and have been used as iPSCs culture substrate (Herron et al., 2016; Kroll et al., 2017).

Synthetic gel and scaffolds are more controllable, reproducible, and exhibit stronger mechanical properties compare to native derived materials, however, they do not have sufficient bioactivity and biocompatibility. Thus, the combinations of native and synthetic polymers are adopted to achieve both biocompatibility and mechanical support in iPSCs based tissue engineering and other applications. For instance, the enzymatically crosslinked poly(ethylene glycol) (PEG)-based hydrogel, which was further modified with fibronectin-derived adhesion peptide Arginne-glycine-aspartate-serine-proline (RGDSP), has been proved that it can boost the hiPSC reprogramming efficacy (Caiazzo et al., 2016). In iPSC based bone tissue engineering, hydroxyapatite-coated poly(lactic-co-glycolic acid)/poly(L-lactic acid) (HA-PLGA/PLLA) scaffolds combined with cultivation of osteoblasts and osteoclasts, which were differentiated from hiPSC-mesenchymal stem cells and macrophages, respectively, resulted in accelerated bone formation both *in vitro* and *in vivo* (Jeon et al., 2016).

### Key Factors in Scaffold Design

Scaffold design for iPSCs related application has to meet the needs of native tissue development and maturation. Several features of engineered scaffold, such as the surface modification, stiffness, topography, and bioactivity, affect the cellular behaviors like attachment, proliferation and differentiation, and the tissue formation.

#### Surface Modification

The surface of biomaterial scaffold can be modified with many functional groups (e.g., -OEG, CH_3_, -OH, -NH_2_, and -COOH), which provide a wide range of wettability and charge (Hao et al., 2016). Hydrophilic surface exhibits greater cell adhesion and migration compared with hydrophobic surface, because hydrophilia enhances the deposition of bioactive ECM proteins, which provides structure motifs for cells to bind. Similarly, the surface charge also affects the cell and protein bindings. The positive charge of amine group (-NH_2_) displays greater protein affinity (Keselowsky et al., 2004) and fibroblasts adhesion and growth (Faucheux et al., 2004) compared with the neutral charged -OH and negative charged -COOH (Lindblad et al., 1997).

Beside the surface charge, the conductivity of scaffold started to draw more attention in the application, particularly in the neural and muscular system. Recently, Gelmi et al. reported a electromechanically active scaffold for iPSC based cardiac tissue engineering by using surface coating of polymer polypyrrole (PPy), a conductive polymer, on electrospun PLGA fiber scaffold (Gelmi et al., 2016). Graphene, single layers of carbon atoms with great electrical conductivity, biocompatibility, mechanical strength and high surface area, can be oxidized by oxygen-containing moieties (e.g., -COOH, -O-, -OH) to improve the surface roughness, colloidal stability and hydrophilicity, making it suitable for scaffold surface modification. When coated with graphene oxide-gold nanosheets, a chitosan scaffold gained the conductivity, and can improve hiPSC-derived cardiomyocytes attachment, differentiation and the *in vivo* cardiac contractility (Saravanan et al., 2018), and for neural tissue engineering (Bei et al., 2019). It is clearly a trend that more bio-electroactive scaffolds can be expected in the near future.

#### Stiffness

It was well-established that the matrix stiffness plays an important role during stem cell differentiation. During the *in vitro* culturing of human mesenchymal stem cells (hMSCs), soft matrix can promote neurogenic differentiation (0.1–1 kPa), moderate matrix supports the myogenic differentiation (8–17 kPa), while rigid matrix favors the osteogenic differentiation (25–40 kPa) (Engler et al., 2006; Gibson et al., 2006). The theory also applied to other stem cells, for instance, the purity and yield of functional motor neurons differentiated from hESC/hiPSCs depends on the rigidity of substrate, soft substrate promote neuroepithelial induction of hiPSCs (Sun et al., 2014). In addition, Kim et al. used fibroblast-derived matrix (FDM) as substrate to culture hiPSCs, and use different concentration of genepin to regulate the biophysical features of the FDM substrate. The FDM showed a range of the Young's modulus at ~100, 800, 5,600, and 8,900 Pa before and after crosslink treatments, and as the stiffness increase, the substrate conserve the pluripotent characteristics of hiPSCs at the expense of growth and migration (Kim et al., 2018). Therefore, the stiffness of biomaterials in iPSCs application should be carefully studied.

#### Scaffold Topography

Topography provides different physical cues in macro-/micro-patterns that regulate the stem cell fate and tissue structure. For example, in an electrospun porous membrane, there are at least three types of topography features that can affect the attached cells—the fiber diameter and direction (Christopherson et al., 2009; Mohtaram et al., 2015), the average pore size of the membrane (Levenberg et al., 2003), and the surface nanosized topography patterns (Abagnale et al., 2017). The fiber diameter and surface nanostructure influence the attached cells, and the pore size of the membrane affect the volume of cells that can fill the scaffold.

Fiber diameter and direction in scaffold affect the attached cell behavior, cells on small fibers showed stretched and multi-directional shape, whereas the cell extension on fibers with larger diameter is restricted. Cooper et al. reported that scaffold topography regulated iPSCs differentiation and showed that larger diameter (400 nm) aligned fibers enhanced iPSCs to become neural cells, while smaller diameter (200 nm) of the fibers promotes the expression of osteogenic and hepatic makers during iPSCs differentiation (Cooper et al., 2012).

The pore shape and size within scaffolds are important cues too. Ji et al. (2015) suggested that sphere-shaped pores give more support for iPSC osteogenic differentiation compared to rod-shaped pores within a nano-hydroxyapatite (HA)/chitosan/gelatin 3D porous scaffold. Meanwhile, Worthington et al. (2016) reported that greater proliferation and neurogenetic differentiation of iPSCs in porous PLGA scaffold.

Besides the macropatterns, the surface micro- and nano-sized features can affect the cell elongation, alignment, migration, polarization, and differentiation (Kong and Mooney, 2007). For example, surface micropatterned nanoridge could induce neurogenic cell morphology, while cells in smooth surface prefer to exhibit fibroblast-like morphology of MSCs (D'Angelo et al., 2010). In sum, topography of scaffold has strong influence on the attached hiPSCs and the hiPSCs based structures, and design the topography of scaffold for hiPSCs depends on the aim of application.

#### Scaffold Bioactivity

ECM compositions and growth factors are usually used to enhance the bioactivity of scaffold in hiPSCs related applications. For example, decellularized fibroblasts-derived matrices can support the maintenance and differentiation of hiPSCs (Lim et al., 2013; Kim et al., 2018), which may be mediated through the activation of integrin and downstream signaling events (Dickinson et al., 2011). Another example is that gelatin scaffold (GS) combined with BMP4 facilitated odontoblastic differentiation of hiPSCs (Ozeki et al., 2017). Encapsulating basic fibroblast growth factor (bFGF) in electrospun polycaprolactone-polyvinylidene fluoride (PCL-PVDF) nanofibrous scaffold can also significantly increase proliferation and osteogenic differentiation of the iPSCs on the scaffold (Abazari et al., 2019).

There are more comprehensive application examples for scaffold design, which the bioactivities were rebuilt based on the requests of spatiality and temporality features of hiPSCs. Xu et al. developed electrospun silk fibroin(SF)/poly(L-lactic acid-co-ε-caprolactone) (PLCL) core-shell fibers for dual-delivering the connective tissue growth factor(CTGF)-derived osteoinductive peptide H1 from the core, and HA from the shell. The resultant dual factor-containing scaffold markedly enhanced adhesion, proliferation and osteoblastic differentiation of hiPSC-derived mesenchymal stem cells (hiPSC-MSCs) (Xu et al., 2019). Mulyasasmita et al. (2014) designed and fabricated protein-polyethylene glycol (PEG) hybrid hydrogels as a tunable injectable carrier for co-delivery of vascular endothelial growth factor (VEGF) and hiPSC-endothelial cells.

These findings collectively suggest the great potential and significance of using biomaterial in iPSCs studies.

## Biofabrication Techniques Used in iPSCs Related Application

### Scaffold Fabrication Techniques

The biofabrication techniques used in manufacturing scaffolds for iPSCs, particularly in hiPSCs based tissue engineering, include the basic solvent casting, gas foaming, lyophilization, phase separation, self-assembling, and more advance, the electrospinning and 3D bioprinting (Figure 1, right panel). Selection of biofabrication technique(s) depends on the nature of biomaterials, and the structural and biological requests of the final products.

Acellularized tissue scaffold is one attractive bioactive scaffold type for iPSC-based tissue engineering because they can reserve the basic structure and bioactivity of the native tissue. hiPSCs cultured on decellularized human brain tissue-derived ECM showed enhanced differentiation into myelin-expressing oligodendrocytes, which involved in many neural disorders (Cho et al., 2019). Electrospun brain decellularized ECM (dECM) nanofibrous scaffold promoted maturation of hiPSC derived oligodendrocytes with increased production of myelin sheath-like structures (Cho et al., 2019). dECM have also been successfully used as cell-laden bio-ink (Pati and Cho, 2017). Yu et al. (2019) demonstrated the potential of using dECM of heart and liver in hiPSC 3D bioprinting. However, the derivation, donor sources, and inconsistency between batches make it is hard to refine the acellularized tissue scaffolds and dECM, which are crucial factors in manufacturing. Using polymers and other biomaterials for scaffold fabrication is more consistent and controllable, and the scaffold geometry structure can be precisely controlled from the general frame to very detailed surface nano-patterns by solvent casting (Li and Wurster, 2018), electrospinning (Mohtaram et al., 2015), lyophilization (Ji et al., 2016), surface treatments (Abagnale et al., 2017), etc.

Most of these methods can produce scaffolds with high porosity and controllable pore size and structure, however, there are limitations of each method. The classic techniques of solvent casting and gas forming bring inadequate pore interconnectivity, that limits the thickness of scaffolds in application, while the freeze drying has temperature requirements when manufacturing. Multiple types of biomaterials and biofabrication techniques are thus used in combination when re-establish the complex tissues and organs, such as for bone, neuron, liver, and heart tissue engineering. For example, a polymeric nanofibrous constructs made by hydroxyapatite/chitosan/gelatin with high porosity can be manufactured by phase separation technique based on the natural of materials (Ji et al., 2016). More advanced technique is electrospinning, with the combinations of using natural polymers (e.g., collagen, gelatin, chitosan, and dECM) and synthetic polymers (e.g., PLLA, PCL), has been applied in building scaffolds for iPSCs based neuron, liver, heart, bladder, pancreas, bone, cartilage, and tendon tissues. Recently, the 3D bioprinting is a burgeoning technology with higher accuracy and greater shape complexity in biomaterials-based scaffold fabrication. Moreover, living cells also can be printed with 3D printing in bio-inks. As reported, biomaterials such as alginate, cellulose, chitosan, agarose (Gu et al., 2017; Nguyen et al., 2017), PEG (Maiullari et al., 2018), hyaluronic acid and gelatin methacrylate (GelMA) (Ma et al., 2016) have been used bio-inks in iPSC-based 3D printing. Table 1 summarized the typical examples of classic and advanced scaffold fabrication technologies that are used in iPSC-based tissue engineering applications, and their advantages and disadvantages (Table 1).

**Table 1:** Biofabrication techniques used in hiPSC-based tissue engineering: cardiac, neural tissue, and others.

**Fabrication technique**	**Biomaterials used in hiPSC-based tissue engineering (TE)**	**Advantage**	**Disadvantage**
Solvent casting/Particulate leaching	• Alginate/chitosan/gelatin in TE of neuron (Kuo and Hsueh, 2017) • PLGA in TE of neural retina (Worthington et al., 2016) • Polyurethane in TE of vascular tissue (Lin et al., 2017) • PCL/borophosphosilicate glass/boron in TE of bone (Mondal et al., 2019)	• High porosity	• Produce thin membranes up to 3 mm thick • Unable to control individual pore structure and distribution
Gas foaming	• Applicable biomaterials such as silk fibroin (Maniglio et al., 2018), cellulose (Lee et al., 2015), and collagen (Croutze et al., 2013) • These biomaterials are suitable for iPSC-based tissue engineering, can be used in hiPSC tissue engineering in the future	• Free of organic solvents • Encapsulation of bioactive species	• Inadequate pore interconnectivity • Unable to control pore structure and distribution
Freeze drying	• Methacrylate-modified HA (HAMA) in Rett syndrome disease modeling of neuron (Zhang et al., 2016)	• Pore diameter and porosity in the scaffolds can be regulated • Keep bioactivity of proteins and peptides	• Cannot engineer scaffolds with hierarchical structures
Phase separation	• Hydroxyapatite/chitosan/gelatin used in TE of bone (Ji et al., 2015)	• Produce polymeric nanofibrous constructs • Produce high porosity and interconnected scaffolds • Stable between batches	• Limited materials combinations can use this method • Inadequate resolution • No orientation or alignment of the scaffold • Less control over fiber diameter (Ma and Zhang, 1999)
Self-assembly	• Collagen/HA/alginate, and the peptides of fibronectin fragment GRGDSP and laminin fragment Ln5-P4, were self-assembled as scaffold for induce differentiation of hiPSCs and TE of neuron (Kuo and Hsueh, 2017)	• Assemble scaffold without directed external intervention • Biomimetic, bioactive • Widely used in protein, peptide, hydrogel • Cells and bioactive agents can be incorporated	• Limited mechanical property and stability • No orientation or alignment of the scaffold • Case-by-case, depends on the properties of the precursor monomers, the specific intra- and intermolecular interactions from molecular identity
Electrospinning	• Polyaniline/polyetersulfone used in TE of heart (Mahmoodinia Maymand et al., 2017) • PLLA used in TE of neuron (Lin et al., 2018) • Electrospun brain dECM nanofibrous scaffold promoted maturation of hiPSC derived oligodendrocytes with increased production of myelin sheath-like structures (Cho et al., 2019) • Collagen/polyethersulfone used in TE of liver (Mahmoodinia Maymand et al., 2017) • HA/collagen/chitosan used in TE of bone (Xie et al., 2016) • PCL/gelatin used in TE of cartilage (Liu et al., 2014) • Chitosan used in TE of tendon (Zhang et al., 2015) • Poly(lactide-co-glycolide) used in TE of bladder (Mirzaei et al., 2019) • Collagen/Polyethersulfon used in TE of pancreas (Mansour et al., 2018)	• Generate ultrafine fibers with diameters ranging from <2 nm to several micrometers (Huang et al., 2006) • High surface area to volume ratio beneficial for cell attachment and bioactive factors loading • Capable of producing long, continuous fibers • Possibility to control fiber morphology (e.g., porous or core-shell) • Apply to plethora of polymers • Avoid temperature changing • Modified into electroblowing avoiding use of solvent, and electrospraying to form thin layer of polymer	• Limited control of pore structures • Process depends on many variables, such as solution, processing parameters, and atmosphere • Sometimes used solvents like surfactants can be toxic
3D bioprinting	• Alginate and PEG-Fibrinogen used in TE of heart (Maiullari et al., 2018) • Using dECM of heart and liver tissue in hiPSC 3D bioprinting (Yu et al., 2019) • Alginate/chitosan/agarose used in TE of neuron (Gu et al., 2017) • GelMA/GMHA used in TE of liver (Ma et al., 2016) • HA used in TE of liver (Ma et al., 2016) • Cellulose/Alginate used in TE of cartilage (Nguyen et al., 2017)	• Automated, high accuracy, controllable, and reproducible • Covers a broad range of biomaterials • Cells and bioactive agents can be incorporated • High resolution • Fabricate constructs of multicellular, automical architecture providing vasculature	• Cell viability can be affected while applying extrusion pressure • The size of objects is limited • The current resolution is insufficient to print capillaries, which are as small as 3 μm in diameter (Potter and Groom, 1983)

### Cell Self-Assembling Techniques

Besides the scaffold, the cell based self-assembling techniques are used abundantly in iPSCs based tissue engineering. The typical one is cell-sheet technology, which was firstly derived from the monolayer cell cultures with enriched ECM production to form a sheet spontaneously. Later on, multiple cell-sheets are folded into 3D tissue structures. This technology gradually progressed with cells seeded on the dishes which are modified with temperature-responsive polymer poly(*N*-isopropylacrylamide) (PIPAAm) at a nanometer-scale thickness, or coated with native ECM derived gels (e.g., type I collagen, fibrin), that can further be easily removable from the petridish by changing temperature or treatment with enzymes, respectively (Figure 1, middle panel, B). The application of cell-sheet technology is not limited to single cell type, multiple-linage co-cultures can also be introduced when the cell sheets stack together to mimic the structure of the native tissue. The cell-sheet technology is currently limited in thickness of reconstructed tissue due to insufficient oxygen and nutrient supply to the core, but iPSC-based cell-sheet has been used for thin tissue regeneration, such as in the retina clinical trial (Mandai et al., 2017). Furthermore, the origami-based smart scaffolds are the further development of cell-sheet technology (Kim et al., 2015). This fabrication process relies on computer-aided designs of the 3D scaffold structure, which can control the internal stresses within scaffolds, and transform the scaffold sheets into designed 3D structures, thus enhance the complexity and robustness of the original cell-sheets (Kim et al., 2015).

Organoid is another representative self-assembly biofabrication technique that is well-used in iPSCs based tissue engineering (Figure 1, middle panel, A). Organoids are the tiny organ like-3D tissue structures, which can be derived from pluripotent stem cells following the developmental processes *in vitro* (McCauley and Wells, 2017). It could represent the complex physiological features of organs or tissues during both normal development and disease affected changes. The first successfully established organoid is intestinal organoid in 2009 (Sato et al., 2009). Hans Clevers and his colleagues used single Lgr5^+^ stem cell to generate crypt-villus organoids without epithelial cellular niche (Sato et al., 2009). Afterwards, synthetic hydrogel were introduced into the culture system, which can improve the formation of organoids. Glorevske et al., reported that PEG hydrogel backbone functionalized with RGD(Arg-Gly-Asp) is sufficient for dynamic control of the culture condition to improve the proliferation and differentiation of intestinal stem cells and organoids (Glorevski et al., 2016; Gjorevski and Lutolf, 2017). The biomaterials assisted iPSCs-based organoids thus provide a more flexible cell model for almost any differentiation requirement for future applications.

Although cell-based self-assembling methods have been widely used in tissue engineering, the tissue size problem remained, the maximum size/thickness of the 3D structure is limited by the oxygen and nutrient diffusion, and manufacturing bigger/thicker tissues may result in necrotic cores. As improvement, combining the self-assembled small tissue units with blood supply and/or ventilation channels by using designed scaffolds and bioreactors probably could overcome the size limitation by providing a pathway for nutrients and oxygen supply.

## Biomaterials in hiPSCs Based Disease Modeling

Lately, patient-derived iPSCs have been used to model colonic tissues for drug screening (Crespo et al., 2017). HiPSCs derived lung tissues, including human alveoli and lung bud organoids have been established to model pulmonary tissues (Chen et al., 2017; Yamamoto et al., 2017). Therefore, it is gradually proven that hiPSCs can be used in disease modeling and drug screening, specifically for those caused by genetic mutations, and lacking appropriate animal models.

Central nerve system is one of the most complex system in human. Scientists have come up with strategies to modeling forebrain spheroid from hiPSCs with functional cortical neurons (Birey et al., 2017). Moreover, using bioreactors, hiPSCs derived specific region of brain tissue could be modeled within several months (Qian et al., 2018). The brain organoids could also be vascularized through re-embedded in Matrigel with hiPSC-derived endothelial cells, which can be transplanted into animals to form human CD31^+^ blood vessels within the organoid (Pham et al., 2018). The development of brain organoids is valuable for studying complex neural development and disease (Logan et al., 2019). Similar applications of hiPSCs derived organoids were reported for heart tissues and disease (Hoang et al., 2018).

In this session, biomaterials used in patient derived iPSCs based disease modeling with genetic mutation in neuromuscular disease (e.g., skeletal muscle, cardiac muscle, and neurons; Figure 1, left panel) are discussed.

### Duchenne Muscular Dystrophy

Dystrophin is a cytoplasmic protein in the linker protein complex that connect cytoskeleton of muscle fibers to local ECM. Mutation of dystrophin gene can cause the DMD, a severer degenerative muscle disorder that affects mostly boys in early childhood, progress to disability at around 12 years old, and the average life expectancy is 26 years old. Because animal models and human conditions are different in DMD, tissue engineering a three-dimensional artificial skeletal muscle tissue from DMD patient derived hiPSCs (Choi et al., 2016) become a potential powerful model for studying the pathology and therapy.

In order to generate muscle tissue *in vitro*, the first step is to differentiate the hiPSCs into skeletal muscle cells. The myogenesis protocol of hiPSCs is a step-wise differentiation protocol. Firstly, the hiPSCs is differentiated into myogenic progenitors with factors such as Pax3/Pax7, and followed by the myogenic regulatory factor (MRF) MyoD/Myf5/Mrf4 to get myoblasts, then use Myogenin to get mature myotubes (Kodaka et al., 2017). After the hiPSCs were fully differentiated in to mature myotubes, these cells would be able to build 3D skeletal muscle tissue for drug screening (Uchimura et al., 2017).

Several ECM proteins have been adopted as scaffold for skeletal muscle tissue engineering, such as collagen, fibrin and laminin (Juhas et al., 2016; Shadrin et al., 2016), which has the potential to be used in hiPSCs skeletal muscle tissue engineering. For example, during the differentiation and the assembling of myotubes from iPSCs, fibrin gel is used to embed the differentiated myoprogenitors, which eventually form aligned multi-nucleated myotubes that exhibit calcium transients in response to electrical stimulation (Rao et al., 2018). Supplementing Matrigel during muscle formation can improve the engineered muscle structure, as myofibers in healthy muscle directly interact with basal lamina proteins (Sato et al., 2011). Nakayama et al. (2018) fabricated 3D parallel aligned nanofibrillar scaffolds with type I collagen, which significantly improved vascular perfusion and muscle innervation after *in vivo* implantation for volumetric muscle loss animals. These successfully applications in muscle tissue engineering suggested that these knowledge are transferable to hiPSC-based muscle tissue engineering for DMD.

A recent sophisticated model for hiPSCs derived DMD muscle tissue has utilized the fibrin hydrogel to combine multiple DMD-hiPSCs derived cell types, which include iPSCs derived myofibers, vascular endothelial cells, pericytes, and motor neurons (Maffioletti et al., 2018). In this multilineage culture system, isogenic hiPSC-derived endothelial cells, pericytes, and myogenic cells were firstly generated and embedded within fibrin hydrogels with uniaxial tension to help the myogenic differentiation. Neurofilament protein SMI32^+^ cells with long axon-like processes spreading from hiPSC-derived neurospheres were further placed above the hydrogels to differentiate from neural precursors into motor neurons. A 3D-hiPSC derived artificial skeletal muscle model of DMD were built and pathological cellular hallmarks can be modeled with high fidelity (Maffioletti et al., 2018).

One interesting application of biomaterials in DMD-hiPSCs is that the nano-topography of cell seeding materials can result different myotube forming patterns of healthy and DMD-hiPSCs, and this myotube alignment differences can provide a sensitive phenotypic readout as a biomarker for related drug testing (Xu et al., 2018). Xu et al. differentiated myotubes from hiPSCs-derived myogenic progenitors with non-diseased, less-affected DMD, and severely-affected DMD, respectively, and further tested the morphologies of myotubes when they were cultured on substrates patterned with nanogrooves. They discovered that myotubes derived from healthy iPSCs aligned almost perpendicular to the nanogrooves, while the counterparts with severely affected DMD showed random orientation, and myotubes from less-affected DMD donors aligned approximately 14° off the alignment direction of non-diseased myotubes. Based on the distinct cell morphologies in alignment and orientation, this disease affected special phenotype of hiPSCs can be used as a simple and cost-effective readout for DMD drug screening, and serves as a complementary tool for early diagnosis of DMD (Xu et al., 2018).

### Cardiac Muscle—Congenital Heart Diseases

Similar to the DMD, genetic mutation caused cardiac muscle abnormalities can be modeled by patient derived hiPSCs. CHDs caused by alteration in the *DAND5* gene, can be represented by iPSCs generated from patients (Cristo et al., 2017). Hypoplastic left heart (HLH) patient derived iPSCs were used to identify primary cardiac defects such as changes in expression of cardiac transcription factors and changes in histone modification (Bosman et al., 2015). Channelopathies can be modeled by iPSCs for cardiometabolic diseases. iPSCs derived from a patient with a heterozygous D1257N mutation in the *SCN5A* gene, which encodes for a subunit in the cardiac voltage-gated sodium channel NaV1.5, were used to show the mutation lowered NaV1.5 levels (Hayano et al., 2017). Cardiomyopathy modeling of left ventricular non-compaction (LVNC) through iPSCs derived from LVNC patients with a mutation in the cardiac transcription factor gene *TBX20* (Kodo et al., 2016), and familial hypertrophic cardiomyopathy (HCM) can be established by patient derived iPSCs with a single missense mutation in the *MYH7* Gene (Han et al., 2014).

To generate a piece of cardiac muscle from hiPSCs, hiPSCs-cardiomyocytes can be seeded in hydrogel-based scaffolds, which are commonly used to mimic the microenvironment of myocardium and form cell-sheet. Natural ECM protein collagen type I, fibronectin, and laminin are commonly used, and PGA, hyaluronic acid (HyA), or mixtures of HyA with alginate also can support the 3D modeling of cardiac muscle (Breckwoldt et al., 2017). In addition to the ECM and hydrogel, electrospun nanofibrous scaffolds are utilized to provide stronger support and 3D structure. Amirabad et al. seeded hiPSCs derived cardiomyocytes onto the electrospun polyaniline/polyetersulfone (PANI/PES) nanofibrous scaffolds modified with camphor-10-sulfonic acid (β) (CPSA), then cultured this 3D structure in cardiomyocyte-inducing factors, and exposed to unidirectional electrical impulse mimicking the unidirectional wave of electrical stimulation like the native cardic tissue, which significantly enhanced the cardiomyocyte differentiation of cardiac patient -specific iPSCs (Mohammadi Amirabad et al., 2017).

Even with the patient specific-hiPSCs derived cardiomyocytes, heart is still a very complex organ to recapitulate. Integration of more cell types, such as cardiac fibroblasts and endothelial cells are crucial in myocardial tissue engineering and disease modeling, and scaffolds are also required for the formation of the complicated 3D structures. Kim et al. demonstrate improved endothelial differentiation of iPSCs when seeded on the electrospun porous PCL microfibrous scaffolds, especially with parallel-aligned fiber orientation, which can further induce anisotropic vascular network-like organization (Kim et al., 2017).

A more advanced fabrication method to incorporate multiple cell types to form comprehensive tissue is the 3D bioprinting technology (Figure 1, right panel, A), which had been used in hiPSCs derived myocardial tissue engineering. HiPSCs based scaffold-free cardiac constructs can be assembled by 3D bioprinter with iPSCs-derived cardiomyocytes, human umbilical vein endothelial cells (HUVECs), and normal human dermal fibroblasts (NHDFs). These cells were suspended and mixed to form cardiac spheroids containing total of 35,000 cells/spheroid, then the spheroids were placed on a needle array by 3D-Bioprinter according to the desired 3D design to form functional tubular cardiac constructs (Arai et al., 2018). More often, bio-inks such as hydrogel and ECMs were applied in the 3D bio-printing of hiPSCs based cardiac tissue. Maiullari et al. used 3D bioprinting in combination with HUVECs and iPSC-derived cardiomyocytes to fabricate a model of vascularized cardiac tissue. In this study, the cells were encapsulated within hydrogel strands containing alginate and PEG-Fibrinogen and extruded through a bioprinter that allows to precisely tailor their 3D spatial deposition, a multi-cellular 3D bioprinted cardiac tissue patch was formed, and the hiPSCs in the scaffold had differentiated into cardiac phenotype with better overall alignment of cardiomyocytes and blood vessels ingrowth in the 3D printed tissue compared to the hiPSC-cardiomyocytes in bulk hydrogel after *in vivo* implantation (Maiullari et al., 2018). Gao et al. produced hiPSC-derived cardiac muscle patch (hCMP) from hiPSC-derived cardiomyocytes, smooth muscle cells, and endothelial cells, and a native ECM structural-like scaffold with the resolution in submicron scale. The native ECM—mimic scaffold was printed via 3D- multiphoton-excited bio-printer with methacrylated gelatin and sodium 4-[2-(4-morpholino)benzoyl-2-dimethylamino]-butylbenzenesulfonate (MBS), with ECM components and patterns that support either cardiomyocytes, endothelial cells or smooth muscle cells in a multilayers structure that to generate hCMPs (Gao et al., 2017).

In future applications, biomaterial scaffold with elastic and electrical conductivity have the potential to be adopted in hiPSC based cardiac tissue engineering, cardiac functional test, and drug screening. The elastic and electrically conductive devices can provide stimulations for tissue maturation, and examine the mechanical and electronical signals of hiPSCs derived myocardial tissue. It has been used in cardiac tissue engineering—a porous conductive scaffold from aniline pentamer-modified polyurethane/PCL blend was made for cardiac tissue engineering (Baheiraei et al., 2015). And more biofabrication methods and applications include the elastic suspension (Godier-Furnemont et al., 2015), elastic silicone posts (Boudou et al., 2012), or thin silicone frames (Jackman et al., 2016) that allow the engineered cardiac tissue to perform contractile work can be expected. One interesting study of using biomaterial-electronics hybrid scaffold to record the electrophysiological activities of attached cells is inspiring for future hiPSCs based application—nanocomposite fibers of polycaprolactone-gelatin were combined with electronic mesh as hybrid scaffold to host the cardiac cells to form a functional cardiac tissue, and the electrophysiological activities can be recorded from the scaffold (Feiner et al., 2016).

### Neurological Diseases Modeling

Patient-derived iPSCs had been utilized to model frontotemporal dementia (FTD), amyotrophic lateral sclerosis (ALS) Alzheimer's disease (AD), *in vitro* for drug and therapeutic screenings (Figure 1A, left panel).

For FTD and ALS, disease specific iPSC-derived cells were used, such as FTD-associated mutation in the *PRGN* gene in hiPSCs astrocytes (Valdez et al., 2017), and a mutation shared by both FTD and ALS, the hexanucleotide GGGGCC repeat expansions in *C9ORF72*, was modeled using iPSC-derived motor neurons (Lopez-Gonzalez et al., 2016).

For AD, astrocytes generated from patient-derived iPSCs can model the early stages of the disease (Jones et al., 2017), and be used for drug screening (Oksanen et al., 2017). Detection of amyloid β peptides (Aβs) in AD-patient iPSC-derived cortical neurons can also be used as an AD assay (Kondo et al., 2017). CRISPR-Cas9 editing correction of the genetic mutation of AD can be tested in hiPSCs based AD modeling. Basal forebrain cholinergic neurons (BFCNs), one of the first cell types to be affected in all forms of AD, were generated from iPSCs derived from patients with the *PSEN2*^*N*141*I*^ mutation, and the correction of the mutation using CRISPR-Cas9 editing led to the abolishment of disease phenotypes (Ortiz-Virumbrales et al., 2017).

The involvement of biomaterials and 3D structure further enhances the effectiveness of AD-hiPSCs based modeling (Yildirimer et al., 2019), e.g., constructing a 3D hydrogel culture with hiPSCs derived neuroepithelial stem cell line AF22 and synthetic peptide RADA-16 can recapitulate the *in vivo*-like responses of AD, such as increased levels of activated p21-activated kinase and their relocation to the submembraneous regions under Aβ oligomer treatment were detected, which is not possible to detect in conventional 2D cultures (Zhang et al., 2014). Matrigel has been used in generating 3D cerebral organoids for AD and FTD modeling due to its capability of developing tau aggregation in AD-iPSCs, which does not happen in 2D culture systems either (Raja et al., 2016; Seo et al., 2017).

## Future Applications

Although significant progresses have been made, few types of scaffolds have reached the pre-clinical or clinical trials. The biocompatibility, long-term stability, and degradation speed, integration to native tissues, immune reactions, and other safety concerns still remain. The biofabrication technologies applied in iPSCs based clinical trials are mostly natural derived materials. For example, in the application of autologous hiPSC-derived retinal pigment epithelium (RPE) in patients to treat exudative age-related macular degeneration (Mandai et al., 2017), cell-sheet was formed with the assistance of porcine derived collagen membrane. The hiPSCs were reprogrammed from patient's own skin fibroblasts, and were differentiated into RPE cells, then the purified RPE cells were seeded on porcine derived collagen, which acts as an artificial scaffold and can be decomposed by collagenase, to obtain a scaffold-free RPE cell-sheet. The autologous 1.3 × 3.0 mm iPSC-derived RPE cell-sheet was then transplanted under the retina of the patient (Mandai et al., 2017). Another example is that ViaCyte and other teams are developing islet cell replacement therapy through subcutaneous implantation of hiPSC-derived pancreatic beta cell progenitors encapsulated in an immunoisolating device in patients with type I/II diabetes, which are mainly alginate based (Strand et al., 2017). These biofabrication methods and biomaterials had been proven safe and effective in other tissue engineering applications, thus are transferable to the hiPSCs based applications. It is naturally to expect that more biomaterials and biofabrication technologies which had been used in other tissue engineering occasions, can be adopted in hiPSCs based applications.

Besides the disease modeling, biomaterial-based biomolecules delivery in a precise and controllable manner is in need for controlling the differentiation and application of hiPSCs. More biofabrication technologies for scaffold manufacturing, drug, and cell delivery including shape-memory elastomeric scaffold fabricated through micromolding technique (Montgomery et al., 2017), and external stimuli (e.g., pH, temperature, and light)-sensitive biomaterial (Knipe and Peppas, 2014) can be expected. Furthermore, hiPSC-biomaterial combination are able to model the physiological and pathological conditions *in vitro* for many other tissue and organs, such as to be applied in studying the reestablishment of the stem cell niche (bone marrow), immune system (lymphatic) (Galat et al., 2017), and nutrient support (blood supply) (Atchison et al., 2017) in the future.

## Author Contributions

JS and YJ contributed with the conception and design. JS, YJ, XM, BF, and HC drafted the manuscript. HC wrote the iPSC-based disease modeling section. XM wrote the organoids section. JS, RT, and YJ revised the manuscript. All authors agree on the final submission of the manuscript.

### Conflict of Interest

The authors declare that the research was conducted in the absence of any commercial or financial relationships that could be construed as a potential conflict of interest.

## References

[B1] Abagnale G. Sechi A. Steger M. Zhou Q. Kuo C. C. Aydin G.. (2017). Surface topography guides morphology and spatial patterning of induced pluripotent stem cell colonies. Stem Cell Rep. 9, 654–666. 10.1016/j.stemcr.2017.06.016 28757164 PMC5550028

[B2] Abazari M. F. Soleimanifar F. Enderami S. E. Nematzadeh M. Nasiri N. Nejati F.. (2019). Incorporated-bFGF polycaprolactone/polyvinylidene fluoride nanocomposite scaffold promotes human induced pluripotent stem cells osteogenic differentiation. J. Cell. Biochem. 120, 16750–16759. 10.1002/jcb.28933 31081968

[B3] Arai K. Murata D. Verissimo A. R. Mukae Y. Itoh M. Nakamura A.. (2018). Fabrication of scaffold-free tubular cardiac constructs using a Bio-3D printer. PLoS ONE 13:e0209162. 10.1371/journal.pone.0209162 30557409 PMC6296519

[B4] Atchison L. Zhang H. Cao K. Truskey G. A. (2017). A tissue engineered blood vessel model of hutchinson-gilford progeria syndrome using human ipsc-derived smooth muscle cells. Sci. Rep. 7:8168. 10.1038/s41598-017-08632-4 28811655 PMC5557922

[B5] Aymard P. Martin D. R. Plucknett K. Foster T. J. Clark A. H. Norton I. T. (2001). Influence of thermal history on the structural and mechanical properties of agarose gels. Biopolymers 59, 131–144. 10.1002/1097-0282(200109)59:3<131::AID-BIP1013>3.0.CO;2-8 11391563

[B6] Baheiraei N. Yeganeh H. Ai J. Gharibi R. Ebrahimi-Barough S. Azami M.. (2015). Preparation of a porous conductive scaffold from aniline pentamer-modified polyurethane/PCL blend for cardiac tissue engineering. J. Biomed. Mater. Res. A 103, 3179–3187. 10.1002/jbm.a.35447 25765879

[B7] Bei H. P. Yang Y. Zhang Q. Tian Y. Luo X. Yang M.. (2019). Graphene-based nanocomposites for neural tissue engineering. Molecules 24:E658. 10.3390/molecules24040658 30781759 PMC6413135

[B8] Birey F. Andersen J. Makinson C. D. Islam S. Wei W. Huber N.. (2017). Assembly of functionally integrated human forebrain spheroids. Nature 545, 54–59. 10.1038/nature22330 28445465 PMC5805137

[B9] Bosman A. Edel M. J. Blue G. Dilley R. J. Harvey R. P. Winlaw D. S. (2015). Bioengineering and stem cell technology in the treatment of congenital heart disease. J. Clin. Med. 4, 768–781. 10.3390/jcm4040768 26239354 PMC4470166

[B10] Boudou T. Legant W. R. Mu A. Borochin M. A. Thavandiran N. Radisic M.. (2012). A microfabricated platform to measure and manipulate the mechanics of engineered cardiac microtissues. Tissue Eng. Part A 18, 910–919. 10.1089/ten.tea.2011.0341 22092279 PMC3338105

[B11] Breckwoldt K. Letuffe-Breniere D. Mannhardt I. Schulze T. Ulmer B. Werner T.. (2017). Differentiation of cardiomyocytes and generation of human engineered heart tissue. Nat. Protoc. 12, 1177–1197. 10.1038/nprot.2017.033 28492526

[B12] Caiazzo M. Okawa Y. Ranga A. Piersigilli A. Tabata Y. Lutolf M. P. (2016). Defined three-dimensional microenvironments boost induction of pluripotency. Nat. Mater. 15, 344–352. 10.1038/nmat4536 26752655

[B13] Chen Y. W. Huang S. X. de Carvalho A. L. R. T. Ho S. H. Islam M. N. Volpi S.. (2017). A three-dimensional model of human lung development and disease from pluripotent stem cells. Nat. Cell Biol. 19, 542–549. 10.1038/ncb3510 28436965 PMC5777163

[B14] Cho A. N. Jin Y. Kim S. Kumar S. Shin H. Kang H. C.. (2019). Aligned brain extracellular matrix promotes differentiation and myelination of human-induced pluripotent stem cell-derived oligodendrocytes. ACS Appl. Mater. Interfaces 11, 15344–15353. 10.1021/acsami.9b03242 30974942

[B15] Choi I. Y. Lim H. Estrellas K. Mula J. Cohen T. V. Zhang Y.. (2016). Concordant but varied phenotypes among duchenne muscular dystrophy patient-specific myoblasts derived using a human ipsc-based model. Cell Rep. 15, 2301–2312. 10.1016/j.celrep.2016.05.016 27239027

[B16] Christopherson G. T. Song H. Mao H. Q. (2009). The influence of fiber diameter of electrospun substrates on neural stem cell differentiation and proliferation. Biomaterials 30, 556–564. 10.1016/j.biomaterials.2008.10.004 18977025

[B17] Cooper A. Leung M. Zhang M. (2012). Polymeric fibrous matrices for substrate-mediated human embryonic stem cell lineage differentiation. Macromol. Biosci. 12, 882–892. 10.1002/mabi.201100269 22648909

[B18] Crespo M. Vilar E. Tsai S. Y. Chang K. Amin S. Srinivasan T.. (2017). Colonic organoids derived from human induced pluripotent stem cells for modeling colorectal cancer and drug testing. Nat. Med. 23, 878–884. 10.1038/nm.4355 28628110 PMC6055224

[B19] Cristo F. Inacio J. M. Rosas G. Carreira I. M. Melo J. B. de Almeida L. P.. (2017). Generation of human iPSC line from a patient with laterality defects and associated congenital heart anomalies carrying a DAND5 missense alteration. Stem Cell Res. 25, 152–156. 10.1016/j.scr.2017.10.019 29136563

[B20] Croutze R. Jomha N. Uludag H. Adesida A. (2013). Matrix forming characteristics of inner and outer human meniscus cells on 3D collagen scaffolds under normal and low oxygen tensions. BMC Musculoskelet Disord. 14:353. 10.1186/1471-2474-14-353 24330551 PMC4029534

[B21] D'Angelo F. Armentano I. Mattioli S. Crispoltoni L. Tiribuzi R. Cerulli G. G.. (2010). Micropatterned hydrogenated amorphous carbon guides mesenchymal stem cells towards neuronal differentiation. Eur. Cell Mater. 20, 231–244. 10.22203/eCM.v020a19 20925022

[B22] Dickinson L. E. Kusuma S. Gerecht S. (2011). Reconstructing the differentiation niche of embryonic stem cells using biomaterials. Macromol. Biosci. 11, 36–49. 10.1002/mabi.201000245 20967797

[B23] Drury J. L. Dennis R. G. Mooney D. J. (2004). The tensile properties of alginate hydrogels. Biomaterials 25, 3187–3199. 10.1016/j.biomaterials.2003.10.002 14980414

[B24] Engler A. J. Sen S. Sweeney H. L. Discher D. E. (2006). Matrix elasticity directs stem cell lineage specification. Cell 126, 677–689. 10.1016/j.cell.2006.06.044 16923388

[B25] Faucheux N. Schweiss R. Lutzow K. Werner C. Groth T. (2004). Self-assembled monolayers with different terminating groups as model substrates for cell adhesion studies. Biomaterials 25, 2721–2730. 10.1016/j.biomaterials.2003.09.069 14962551

[B26] Feaster T. K. Cadar A. G. Wang L. Williams C. H. Chun Y. W. Hempel J. E.. (2015). Matrigel mattress: a method for the generation of single contracting human-induced pluripotent stem cell-derived cardiomyocytes. Circ. Res. 117, 995–1000. 10.1161/CIRCRESAHA.115.307580 26429802 PMC4670592

[B27] Feiner R. Engel L. Fleischer S. Malki M. Gal I. Shapira A.. (2016). Engineered hybrid cardiac patches with multifunctional electronics for online monitoring and regulation of tissue function. Nat. Mater. 15, 679–685. 10.1038/nmat4590 26974408 PMC4900449

[B28] Feng B. Jiang J. Kraus P. Ng J. H. Heng J. C. Chan Y. S.. (2009). Reprogramming of fibroblasts into induced pluripotent stem cells with orphan nuclear receptor Esrrb. Nat. Cell Biol. 11, 197–203. 10.1038/ncb1827 19136965

[B29] Galat Y. Dambaeva S. Elcheva I. Khanolkar A. Beaman K. Iannaccone P. M.. (2017). Cytokine-free directed differentiation of human pluripotent stem cells efficiently produces hemogenic endothelium with lymphoid potential. Stem. Cell Res. Ther. 8:67. 10.1186/s13287-017-0519-0 28302184 PMC5356295

[B30] Gao L. Kupfer M. E. Jung J. P. Yang L. Zhang P. Da Sie Y.. (2017). Myocardial tissue engineering with cells derived from human-induced pluripotent stem cells and a native-like, high-resolution, 3-dimensionally printed scaffold. Circ. Res. 120, 1318–1325. 10.1161/CIRCRESAHA.116.310277 28069694 PMC5392171

[B31] GBD 2015 Disease and Injury Incidence and Prevalence Collaborators (2016). Global, regional, and national incidence, prevalence, and years lived with disability for 310 diseases and injuries, 1990–2015: a systematic analysis for the global burden of disease study 2015. Lancet 388, 1545–1602. 10.1016/S0140-6736(16)31678-6 27733282 PMC5055577

[B32] Gelmi A. Cieslar-Pobuda A. de Muinck E. Los M. Rafat M. Jager E. W. (2016). Direct mechanical stimulation of stem cells: a beating electromechanically active scaffold for cardiac tissue engineering. Adv. Healthc. Mater. 5, 1471–1480. 10.1002/adhm.201600307 27126086

[B33] Gibson M. C. Patel A. B. Nagpal R. Perrimon N. (2006). The emergence of geometric order in proliferating metazoan epithelia. Nature 442, 1038–1041. 10.1038/nature05014 16900102

[B34] Gjorevski N. Lutolf M. P. (2017). Synthesis and characterization of well-defined hydrogel matrices and their application to intestinal stem cell and organoid culture. Nat. Protoc. 12, 2263–2274. 10.1038/nprot.2017.095 28981121

[B35] Glorevski N. Sachs N. Manfrin A. Giger S. Bragina M. E. Ordonez-Moran P. (2016). Designer matrices for intestinal stem cell and organoid culture. Nature 539, 560–564. 10.1038/nature20168 27851739

[B36] Gnecchi M. Stefanello M. Mura M. (2017). Induced pluripotent stem cell technology: toward the future of cardiac arrhythmias. Int. J. Cardiol. 237, 49–52. 10.1016/j.ijcard.2017.03.085 28408106

[B37] Godier-Furnemont A. F. Tiburcy M. Wagner E. Dewenter M. Lammle S. El-Armouche A.. (2015). Physiologic force-frequency response in engineered heart muscle by electromechanical stimulation. Biomaterials 60, 82–91. 10.1016/j.biomaterials.2015.03.055 25985155 PMC4921199

[B38] Grounds M. D. Torrisi J. (2004). Anti-TNFalpha (Remicade) therapy protects dystrophic skeletal muscle from necrosis. FASEB J. 18, 676–682. 10.1096/fj.03-1024com 15054089

[B39] Gu Q. Tomaskovic-Crook E. Wallace G. G. Crook J. M. (2017). 3D bioprinting human induced pluripotent stem cell constructs for in situ cell proliferation and successive multilineage differentiation. Adv. Healthc. Mater. 6:1700175. 10.1002/adhm.201700175 28544655

[B40] Gu Q. Zhu H. Chen L. Shuai L. Fang J. Wu J.. (2016). Three dimensional collagen scaffolds promote iPSC induction with higher pluripotency. Protein Cell 7, 844–848. 10.1007/s13238-016-0321-2 27730497 PMC5084158

[B41] Guo D. Wu F. Liu H. Gao G. Kou S. Yang F.. (2017). Generation of non-integrated induced pluripotent stem cells from a 23-year-old male with multiple endocrine neoplasia type 1 syndrome. Stem Cell Res. 18, 70–72. 10.1016/j.scr.2016.12.002 28395810

[B42] Han L. Li Y. Tchao J. Kaplan A. D. Lin B. Li Y.. (2014). Study familial hypertrophic cardiomyopathy using patient-specific induced pluripotent stem cells. Cardiovasc. Res. 104, 258–269. 10.1093/cvr/cvu205 25209314 PMC4217687

[B43] Hao L. Fu X. Li T. Zhao N. Shi X. Cui F.. (2016). Surface chemistry from wettability and charge for the control of mesenchymal stem cell fate through self-assembled monolayers. Colloids Surf. B Biointerfaces 148, 549–556. 10.1016/j.colsurfb.2016.09.027 27690244

[B44] Hayano M. Makiyama T. Kamakura T. Watanabe H. Sasaki K. Funakoshi S.. (2017). Development of a patient-derived induced pluripotent stem cell model for the investigation of scn5a-d1275n-related cardiac sodium channelopathy. Circ. J. 81, 1783–1791. 10.1253/circj.CJ-17-0064 28637969

[B45] Heng J. C. Feng B. Han J. Jiang J. Kraus P. Ng J. H.. (2010). The nuclear receptor Nr5a2 can replace Oct4 in the reprogramming of murine somatic cells to pluripotent cells. Cell Stem Cell 6, 167–174. 10.1016/j.stem.2009.12.009 20096661

[B46] Herron T. J. Rocha A. M. Campbell K. F. Ponce-Balbuena D. Willis B. C. Guerrero-Serna G.. (2016). Extracellular matrix-mediated maturation of human pluripotent stem cell-derived cardiac monolayer structure and electrophysiological function. Circ. Arrhythm. Electrophysiol. 9:e003638. 10.1161/CIRCEP.113.003638 27069088 PMC4833010

[B47] Hoang P. Wang J. Conklin B. R. Healy K. E. Ma Z. (2018). Generation of spatial-patterned early-developing cardiac organoids using human pluripotent stem cells. Nat. Protoc. 13, 723–737. 10.1038/nprot.2018.006 29543795 PMC6287283

[B48] Huang C. Chen S. Lai C. Reneker D. H. Qiu H. Ye Y.. (2006). Electrospun polymer nanofibres with small diameters. Nanotechnology 17, 1558–1563. 10.1088/0957-4484/17/6/004 26558558

[B49] Jackman C. P. Carlson A. L. Bursac N. (2016). Dynamic culture yields engineered myocardium with near-adult functional output. Biomaterials 111, 66–79. 10.1016/j.biomaterials.2016.09.024 27723557 PMC5074846

[B50] Jeon O. H. Panicker L. M. Lu Q. Chae J. J. Feldman R. A. Elisseeff J. H. (2016). Human iPSC-derived osteoblasts and osteoclasts together promote bone regeneration in 3D biomaterials. Sci. Rep. 6:26761. 10.1038/srep26761 27225733 PMC4881234

[B51] Ji J. Tong X. Huang X. Wang T. Lin Z. Cao Y.. (2015). Sphere-shaped nano-hydroxyapatite/chitosan/gelatin 3D porous scaffolds increase proliferation and osteogenic differentiation of human induced pluripotent stem cells from gingival fibroblasts. Biomed. Mater. 10:045005. 10.1088/1748-6041/10/4/045005 26154827

[B52] Ji J. Tong X. Huang X. Zhang J. Qin H. Hu Q. (2016). Patient-derived human induced pluripotent stem cells from gingival fibroblasts composited with defined nanohydroxyapatite/chitosan/gelatin porous scaffolds as potential bone graft substitutes. Stem Cells Transl. Med. 5, 95–105. 10.5966/sctm.2015-0139 26586776 PMC4704877

[B53] Jones V. C. Atkinson-Dell R. Verkhratsky A. Mohamet L. (2017). Aberrant iPSC-derived human astrocytes in Alzheimer's disease. Cell Death Dis. 8:e2696. 10.1038/cddis.2017.89 28333144 PMC5386580

[B54] Juhas M. Ye J. Bursac N. (2016). Design, evaluation, and application of engineered skeletal muscle. Methods 99, 81–90. 10.1016/j.ymeth.2015.10.002 26455485 PMC4821818

[B55] Keselowsky B. G. Collard D. M. Garcia A. J. (2004). Surface chemistry modulates focal adhesion composition and signaling through changes in integrin binding. Biomaterials 25, 5947–5954. 10.1016/j.biomaterials.2004.01.062 15183609

[B56] Khan F. Tanaka M. (2017). Designing smart biomaterials for tissue engineering. Int. J. Mol. Sci. 19:E17. 10.3390/ijms19010017 29267207 PMC5795968

[B57] Kim I. G. Gil C. H. Seo J. Park S. J. Subbiah R. Jung T. H.. (2018). Mechanotransduction of human pluripotent stem cells cultivated on tunable cell-derived extracellular matrix. Biomaterials 150, 100–111. 10.1016/j.biomaterials.2017.10.016 29035736

[B58] Kim J. J. Hou L. Yang G. Mezak N. P. Wanjare M. Joubert L. M.. (2017). Microfibrous scaffolds enhance endothelial differentiation and organization of induced pluripotent stem cells. Cell Mol. Bioeng. 10, 417–432. 10.1007/s12195-017-0502-y 28936269 PMC5602598

[B59] Kim S. H. Lee H. R. Yu S. J. Han M. E. Lee D. Y. Kim S. Y.. (2015). Hydrogel-laden paper scaffold system for origami-based tissue engineering. Proc. Natl. Acad. Sci. U.S.A. 112, 15426–15431. 10.1073/pnas.1504745112 26621717 PMC4687567

[B60] Kjaer M. (2004). Role of extracellular matrix in adaptation of tendon and skeletal muscle to mechanical loading. Physiol. Rev. 84, 649–698. 10.1152/physrev.00031.2003 15044685

[B61] Knipe J. M. Peppas N. A. (2014). Multi-responsive hydrogels for drug delivery and tissue engineering applications. Regen. Biomater. 1, 57–65. 10.1093/rb/rbu006 26816625 PMC4669007

[B62] Kodaka Y. Rabu G. Asakura A. (2017). Skeletal muscle cell induction from pluripotent stem cells. Stem Cells Int. 2017:1376151. 10.1155/2017/7824614 28529527 PMC5424488

[B63] Kodo K. Ong S. G. Jahanbani F. Termglinchan V. Hirono K. InanlooRahatloo K.. (2016). iPSC-derived cardiomyocytes reveal abnormal TGF-beta signalling in left ventricular non-compaction cardiomyopathy. Nat. Cell. Biol. 18, 1031–1042. 10.1038/ncb3411 27642787 PMC5042877

[B64] Kondo T. Imamura K. Funayama M. Tsukita K. Miyake M. Ohta A.. (2017). iPSC-based compound screening and *in vitro* trials identify a synergistic anti-amyloid beta combination for Alzheimer's disease. Cell Rep. 21, 2304–2312. 10.1016/j.celrep.2017.10.109 29166618

[B65] Kong H. J. Mooney D. J. (2007). Microenvironmental regulation of biomacromolecular therapies. Nat. Rev. Drug Discov. 6, 455–463. 10.1038/nrd2309 17541418

[B66] Kong Y. P. Rioja A. Y. Xue X. Sun Y. Fu J. Putnam A. J. (2018). A systems mechanobiology model to predict cardiac reprogramming outcomes on different biomaterials. Biomaterials 181, 280–292. 10.1016/j.biomaterials.2018.07.036 30096562 PMC6119647

[B67] Kroll K. Chabria M. Wang K. Hausermann F. Schuler F. Polonchuk L. (2017). Electro-mechanical conditioning of human iPSC-derived cardiomyocytes for translational research. Prog. Biophys. Mol. Biol. 130(Pt B), 212–222. 10.1016/j.pbiomolbio.2017.07.003 28688751

[B68] Kumari A. Yadav S. K. Yadav S. C. (2010). Biodegradable polymeric nanoparticles based drug delivery systems. Colloids Surf. B Biointerfaces 75, 1–18. 10.1016/j.colsurfb.2009.09.001 19782542

[B69] Kuo Y. C. Hsueh C. H. (2017). Neuronal production from induced pluripotent stem cells in self-assembled collagen-hyaluronic acid-alginate microgel scaffolds with grafted GRGDSP/Ln5-P4. Mater. Sci. Eng. C Mater. Biol. Appl. 76, 760–774. 10.1016/j.msec.2017.03.133 28482588

[B70] Lee S. Y. Bang S. Kim S. Jo S. Y. Kim B. C. Hwang Y.. (2015). Synthesis and *in vitro* characterizations of porous carboxymethyl cellulose-poly(ethylene oxide) hydrogel film. Biomater. Res. 19:12. 10.1186/s40824-015-0033-3 26331082 PMC4552372

[B71] Levenberg S. Huang N. F. Lavik E. Rogers A. B. Itskovitz-Eldor J. Langer R. (2003). Differentiation of human embryonic stem cells on three-dimensional polymer scaffolds. Proc. Natl. Acad. Sci. U.S.A. 100, 12741–12746. 10.1073/pnas.1735463100 14561891 PMC240688

[B72] Li Y. Wurster D. E. (2018). The Effects of curing and casting methods on the physicochemical properties of polymer films. AAPS PharmSciTech 19, 2740–2749. 10.1208/s12249-018-1113-1 29978291

[B73] Lim M. L. Jungebluth P. Sjoqvist S. Nikdin H. Kjartansdottir K. R. Unger C.. (2013). Decellularized feeders: an optimized method for culturing pluripotent cells. Stem Cells Transl. Med. 2, 975–982. 10.5966/sctm.2013-0077 24167316 PMC3841092

[B74] Lin C. Liu C. Zhang L. Huang Z. Zhao P. Chen R.. (2018). Interaction of iPSC-derived neural stem cells on poly(L-lactic acid) nanofibrous scaffolds for possible use in neural tissue engineering. Int. J. Mol. Med. 41, 697–708. 10.3892/ijmm.2017.3299 29207038 PMC5752187

[B75] Lin Y. Gil C. H. Yoder M. C. (2017). Differentiation, evaluation, and application of human induced pluripotent stem cell-derived endothelial cells. Arterioscler. Thromb. Vasc. Biol. 37, 2014–2025. 10.1161/ATVBAHA.117.309962 29025705

[B76] Lindblad M. Lestelius M. Johansson A. Tengvall P. Thomsen P. (1997). Cell and soft tissue interactions with methyl- and hydroxyl-terminated alkane thiols on gold surfaces. Biomaterials 18, 1059–1068. 10.1016/S0142-9612(97)00029-X 9239468

[B77] Liu J. Nie H. Xu Z. Niu X. Guo S. Yin J.. (2014). The effect of 3D nanofibrous scaffolds on the chondrogenesis of induced pluripotent stem cells and their application in restoration of cartilage defects. PLoS ONE 9:e111566. 10.1371/journal.pone.0111566 25389965 PMC4229072

[B78] Logan S. Arzua T. Canfield S. G. Seminary E. R. Sison S. L. Ebert A. D.. (2019). Studying human neurological disorders using induced pluripotent stem cells: from 2D monolayer to 3D organoid and blood brain barrier models. Compr. Physiol. 9, 565–611. 10.1002/cphy.c180025 30873582 PMC6705133

[B79] Lopez-Gonzalez R. Lu Y. Gendron T. F. Karydas A. Tran H. Yang D.. (2016). Poly(GR) in C9ORF72-related ALS/FTD compromises mitochondrial function and increases oxidative stress and DNA damage in iPSC-derived motor neurons. Neuron 92, 383–391. 10.1016/j.neuron.2016.09.015 27720481 PMC5111366

[B80] Ma P. X. Zhang R. (1999). Synthetic nano-scale fibrous extracellular matrix. J. Biomed. Mater. Res. 46, 60–72. 10.1002/(SICI)1097-4636(199907)46:1<60::AID-JBM7>3.0.CO;2-H 10357136

[B81] Ma X. Qu X. Zhu W. Li Y. S. Yuan S. Zhang H.. (2016). Deterministically patterned biomimetic human iPSC-derived hepatic model via rapid 3D bioprinting. Proc. Natl. Acad. Sci. U.S.A. 113, 2206–2211. 10.1073/pnas.1524510113 26858399 PMC4776497

[B82] Maffioletti S. M. Sarcar S. Henderson A. B. H. Mannhardt I. Pinton L. Moyle L. A.. (2018). Three-dimensional human iPSC-derived artificial skeletal muscles model muscular dystrophies and enable multilineage tissue engineering. Cell Rep. 23, 899–908. 10.1016/j.celrep.2018.03.091 29669293 PMC5917451

[B83] Mahmoodinia Maymand M. Soleimanpour-Lichaei H. R. Ardeshirylajimi A. Soleimani M. Mirzaei S. Hajarizadeh A.. (2017). Hepatogenic differentiation of human induced pluripotent stem cells on collagen-coated polyethersulfone nanofibers. ASAIO J. 63, 316–323. 10.1097/MAT.0000000000000469 27861428

[B84] Maiullari F. Costantini M. Milan M. Pace V. Chirivì M. Maiullari S.. (2018). A multi-cellular 3D bioprinting approach for vascularized heart tissue engineering based on HUVECs and iPSC-derived cardiomyocytes. Sci. Rep. 8:13532. 10.1038/s41598-018-31848-x 30201959 PMC6131510

[B85] Mandai M. Watanabe A. Kurimoto Y. Hirami Y. Morinaga C. Daimon T.. (2017). Autologous induced stem-cell-derived retinal cells for macular degeneration. N. Engl. J. Med. 376, 1038–1046. 10.1056/NEJMoa1608368 28296613

[B86] Maniglio D. Bonani W. Migliaresi C. Motta A. (2018). Silk fibroin porous scaffolds by N2O foaming. J. Biomater. Sci. Polym. Ed. 29, 491–506. 10.1080/09205063.2018.1423811 29297760

[B87] Mansour R. N. Soleimanifar F. Abazari M. F. Torabinejad S. Ardeshirylajimi A. Ghoraeian P.. (2018). Collagen coated electrospun polyethersulfon nanofibers improved insulin producing cells differentiation potential of human induced pluripotent stem cells. Artif. Cells Nanomed. Biotechnol. 46 (suppl 3), S734–S739. 10.1080/21691401.2018.1508031 30284483

[B88] Martino S. D'Angelo F. Armentano I. Kenny J. M. Orlacchio A. (2012). Stem cell-biomaterial interactions for regenerative medicine. Biotechnol. Adv. 30, 338–351. 10.1016/j.biotechadv.2011.06.015 21740963

[B89] McCauley H. A. Wells J. M. (2017). Pluripotent stem cell-derived organoids: using principles of developmental biology to grow human tissues in a dish. Development 144, 958–962. 10.1242/dev.140731 28292841 PMC5358106

[B90] Mirzaei A. Saburi E. Islami M. Ardeshirylajimi A. Omrani M. D. Taheri M.. (2019). Bladder smooth muscle cell differentiation of the human induced pluripotent stem cells on electrospun Poly(lactide-co-glycolide) nanofibrous structure. Gene 694, 26–32. 10.1016/j.gene.2019.01.037 30735717

[B91] Moat S. J. Bradley D. M. Salmon R. Clarke A. Hartley L. (2013). Newborn bloodspot screening for Duchenne muscular dystrophy: 21 years experience in Wales (UK). Eur. J. Hum. Genet. 21, 1049–1053. 10.1038/ejhg.2012.301 23340516 PMC3778339

[B92] Mohammadi Amirabad L. Massumi M. Shamsara M. Shabani I. Amari A. Mossahebi Mohammadi M.. (2017). Enhanced cardiac differentiation of human cardiovascular disease patient-specific induced pluripotent stem cells by applying unidirectional electrical pulses using aligned electroactive nanofibrous scaffolds. ACS Appl. Mater. Interfaces 9, 6849–6864. 10.1021/acsami.6b15271 28116894

[B93] Mohtaram N. K. Ko J. King C. Sun L. Muller N. Jun M. B.. (2015). Electrospun biomaterial scaffolds with varied topographies for neuronal differentiation of human-induced pluripotent stem cells. J. Biomed. Mater. Res. A 103, 2591–2601. 10.1002/jbm.a.35392 25524598

[B94] Mondal D. Lin S. Rizkalla A. S. Mequanint K. (2019). Porous and biodegradable polycaprolactone-borophosphosilicate hybrid scaffolds for osteoblast infiltration and stem cell differentiation. J. Mech. Behav. Biomed. Mater. 92, 162–171. 10.1016/j.jmbbm.2019.01.011 30710831

[B95] Montgomery M. Ahadian S. Davenport Huyer L. Lo Rito M. Civitarese R. A. Vanderlaan R. D.. (2017). Flexible shape-memory scaffold for minimally invasive delivery of functional tissues. Nat. Mater. 16, 1038–1046. 10.1038/nmat4956 28805824

[B96] Mulyasasmita W. Cai L. Dewi R. E. Jha A. Ullmann S. D. Luong R. H.. (2014). Avidity-controlled hydrogels for injectable co-delivery of induced pluripotent stem cell-derived endothelial cells and growth factors. J. Control Release 191, 71–81. 10.1016/j.jconrel.2014.05.015 24848744 PMC4518026

[B97] Nakayama K. H. Alcazar C. Yang G. Quarta M. Paine P. Doan L.. (2018). Rehabilitative exercise and spatially patterned nanofibrillar scaffolds enhance vascularization and innervation following volumetric muscle loss. NPJ Regen. Med. 3:16. 10.1038/s41536-018-0054-3 30245849 PMC6141593

[B98] Nguyen D. Hagg D. A. Forsman A. Ekholm J. Nimkingratana P. Brantsing C.. (2017). Cartilage tissue engineering by the 3D bioprinting of iPS cells in a nanocellulose/alginate Bioink. Sci. Rep. 7:658. 10.1038/s41598-017-00690-y 28386058 PMC5428803

[B99] Oksanen M. Petersen A. J. Naumenko N. Puttonen K. Lehtonen S. Gubert Olive M.. (2017). PSEN1 mutant iPSC-derived model reveals severe astrocyte pathology in Alzheimer's disease. Stem Cell Rep. 9, 1885–1897. 10.1016/j.stemcr.2017.10.016 29153989 PMC5785689

[B100] Ortiz-Virumbrales M. Moreno C. L. Kruglikov I. Marazuela P. Sproul A. Jacob S.. (2017). CRISPR/Cas9-Correctable mutation-related molecular and physiological phenotypes in iPSC-derived Alzheimer's PSEN2 (N141I) neurons. Acta Neuropathol. Commun. 5:77. 10.1186/s40478-017-0475-z 29078805 PMC5660456

[B101] Ozeki N. Hase N. Higuchi N. Hiyama T. Yamaguchi H. Kawai R.. (2017). Gelatin scaffold combined with bone morphogenetic protein-4 induces odontoblast-like cell differentiation involving integrin profile changes, autophagy-related gene 10, and Wnt5 sequentially in human induced pluripotent stem cells. Differentiation 93, 1–14. 10.1016/j.diff.2016.09.002 27639333

[B102] Park I. H. Arora N. Huo H. Maherali N. Ahfeldt T. Shimamura A.. (2008). Disease-specific induced pluripotent stem cells. Cell 134, 877–886. 10.1016/j.cell.2008.07.041 18691744 PMC2633781

[B103] Pati F. Cho D. W. (2017). Bioprinting of 3D tissue models using decellularized extracellular matrix Bioink. Methods Mol. Biol. 1612, 381–390. 10.1007/978-1-4939-7021-6_27 28634957

[B104] Pen A. E. Jensen U. B. (2017). Current status of treating neurodegenerative disease with induced pluripotent stem cells. Acta Neurol. Scand. 135, 57–72. 10.1111/ane.12545 26748435

[B105] Pham M. T. Pollock K. M. Rose M. D. Cary W. A. Stewart H. R. Zhou P.. (2018). Generation of human vascularized brain organoids. Neuroreport 29, 588–593. 10.1097/WNR.0000000000001014 29570159 PMC6476536

[B106] Potter R. F. Groom A. C. (1983). Capillary diameter and geometry in cardiac and skeletal muscle studied by means of corrosion casts. Microvasc. Res. 25, 68–84. 10.1016/0026-2862(83)90044-4 6835100

[B107] Qian X. Y. Jacob F. Song M. M. Nguyen H. N. Song H. J. Ming G. L. (2018). Generation of human brain region-specific organoids using a miniaturized spinning bioreactor. Nat. Protoc. 13, 565–580. 10.1038/nprot.2017.152 29470464 PMC6241211

[B108] Qu Y. Zhou B. Yang W. Han B. Yu-Rice Y. Gao B.. (2016). Transcriptome and proteome characterization of surface ectoderm cells differentiated from human iPSCs. Sci. Rep. 6:32007. 10.1038/srep32007 27550649 PMC4994084

[B109] Raja W. K. Mungenast A. E. Lin Y. T. Ko T. Abdurrob F. Seo J.. (2016). Self-organizing 3D human neural tissue derived from induced pluripotent stem cells recapitulate Alzheimer's disease phenotypes. PLoS ONE 11:e0161969. 10.1371/journal.pone.0161969 27622770 PMC5021368

[B110] Rao L. Qian Y. Khodabukus A. Ribar T. Bursac N. (2018). Engineering human pluripotent stem cells into a functional skeletal muscle tissue. Nat. Commun. 9:126. 10.1038/s41467-017-02636-4 29317646 PMC5760720

[B111] Saravanan S. Sareen N. Abu-El-Rub E. Ashour H. Sequiera G. L. Ammar H. I.. (2018). Graphene oxide-gold nanosheets containing chitosan scaffold improves ventricular contractility and function after implantation into infarcted heart. Sci. Rep. 8:15069. 10.1038/s41598-018-33144-0 30305684 PMC6180127

[B112] Sato M. Ito A. Kawabe Y. Nagamori E. Kamihira M. (2011). Enhanced contractile force generation by artificial skeletal muscle tissues using IGF-I gene-engineered myoblast cells. J. Biosci. Bioeng. 112, 273–278. 10.1016/j.jbiosc.2011.05.007 21646045

[B113] Sato T. Vries R. G. Snippert H. J. van de Wetering M. Barker N. Stange D. E.. (2009). Single Lgr5 stem cells build crypt-villus structures *in vitro* without a mesenchymal niche. Nature 459, 262–265. 10.1038/nature07935 19329995

[B114] Schwick H. G. Heide K. (1969). Immunochemistry and immunology of collagen and gelatin. Bibl. Haematol. 33, 111–125. 10.1159/000384833 4988117

[B115] Seo J. Kritskiy O. Watson L. A. Barker S. J. Dey D. Raja W. K.. (2017). Inhibition of p25/Cdk5 attenuates tauopathy in mouse and iPSC models of frontotemporal dementia. J. Neurosci. 37, 9917–9924. 10.1523/JNEUROSCI.0621-17.2017 28912154 PMC5637118

[B116] Shadrin I. Y. Khodabukus A. Bursac N. (2016). Striated muscle function, regeneration, and repair. Cell Mol. Life Sci. 73, 4175–4202. 10.1007/s00018-016-2285-z 27271751 PMC5056123

[B117] Spencer M. J. Tidball J. G. (1996). Calpain translocation during muscle fiber necrosis and regeneration in dystrophin-deficient mice. Exp. Cell Res. 226, 264–272. 10.1006/excr.1996.0227 8806430

[B118] Strand B. L. Coron A. E. Skjak-Braek G. (2017). Current and future perspectives on alginate encapsulated pancreatic islet. Stem Cells Transl. Med. 6, 1053–1058. 10.1002/sctm.16-0116 28186705 PMC5442831

[B119] Sun Y. Yong K. M. Villa-Diaz L. G. Zhang X. Chen W. Philson R.. (2014). Hippo/YAP-mediated rigidity-dependent motor neuron differentiation of human pluripotent stem cells. Nat. Mater. 13, 599–604. 10.1038/nmat3945 24728461 PMC4051885

[B120] Takahashi K. Tanabe K. Ohnuki M. Narita M. Ichisaka T. Tomoda K.. (2007). Induction of pluripotent stem cells from adult human fibroblasts by defined factors. Cell 131, 861–872. 10.1016/j.cell.2007.11.019 18035408

[B121] Takahashi K. Yamanaka S. (2006). Induction of pluripotent stem cells from mouse embryonic and adult fibroblast cultures by defined factors. Cell 126, 663–676. 10.1016/j.cell.2006.07.024 16904174

[B122] Tondera C. Hauser S. Kruger-Genge A. Jung F. Neffe A. T. Lendlein A.. (2016). Gelatin-based hydrogel degradation and tissue interaction *in vivo*: insights from multimodal preclinical imaging in immunocompetent nude mice. Theranostics 6, 2114–2128. 10.7150/thno.16614 27698944 PMC5039684

[B123] Uchimura T. Otomo J. Sato M. Sakurai H. (2017). A human iPS cell myogenic differentiation system permitting high-throughput drug screening. Stem Cell Res. 25, 98–106. 10.1016/j.scr.2017.10.023 29125995

[B124] Valdez C. Wong Y. C. Schwake M. Bu G. Wszolek Z. K. Krainc D. (2017). Progranulin-mediated deficiency of cathepsin D results in FTD and NCL-like phenotypes in neurons derived from FTD patients. Hum. Mol. Genet. 26, 4861–4872. 10.1093/hmg/ddx364 29036611 PMC5886207

[B125] Vandenburgh H. H. Karlisch P. Farr L. (1988). Maintenance of highly contractile tissue-cultured avian skeletal myotubes in collagen gel. In Vitro Cell Dev. Biol. 24, 166–174. 10.1007/BF02623542 3350785

[B126] Wheelton A. Mace J. Khan W. S. Anand S. (2016). Biomaterials and fabrication to optimise scaffold properties for musculoskeletal tissue engineering. Curr. Stem Cell Res. Ther. 11, 578–584. 10.2174/1574888X11666160614101037 27306403

[B127] Worthington K. S. Wiley L. A. Guymon C. A. Salem A. K. Tucker B. A. (2016). Differentiation of induced pluripotent stem cells to neural retinal precursor cells on porous poly-lactic-co-glycolic acid scaffolds. J. Ocul. Pharmacol. Ther. 32, 310–316. 10.1089/jop.2015.0126 26692377 PMC4904232

[B128] Xiao X. Li N. Zhang D. Yang B. Guo H. Li Y. (2016). Generation of induced pluripotent stem cells with substitutes for Yamanaka's four transcription factors. Cell Reprogr. 18, 281–297. 10.1089/cell.2016.0020 27696909

[B129] Xie J. Peng C. Zhao Q. Wang X. Yuan H. Yang L.. (2016). Osteogenic differentiation and bone regeneration of iPSC-MSCs supported by a biomimetic nanofibrous scaffold. Acta Biomater. 29, 365–379. 10.1016/j.actbio.2015.10.007 26441129

[B130] Xu B. Magli A. Anugrah Y. Koester S. J. Perlingeiro R. C. R. Shen W. (2018). Nanotopography-responsive myotube alignment and orientation as a sensitive phenotypic biomarker for Duchenne Muscular Dystrophy. Biomaterials 183, 54–66. 10.1016/j.biomaterials.2018.08.047 30149230 PMC6239205

[B131] Xu R. Zhang Z. Toftdal M. S. Moller A. C. Dagnaes-Hansen F. Dong M.. (2019). Synchronous delivery of hydroxyapatite and connective tissue growth factor derived osteoinductive peptide enhanced osteogenesis. J. Control Release 301, 129–139. 10.1016/j.jconrel.2019.02.037 30880079

[B132] Yamamoto Y. Gotoh S. Korogi Y. Seki M. Konishi S. Ikeo S.. (2017). Long-term expansion of alveolar stem cells derived from human iPS cells in organoids. Nat. Methods 14, 1097–1106. 10.1038/nmeth.4448 28967890

[B133] Yildirimer L. Zhang Q. Kuang S. Cheung C. J. Chu K. A. He Y.. (2019). Engineering three-dimensional microenvironments towards *in vitro* disease models of the central nervous system. Biofabrication 11:032003. 10.1088/1758-5090/ab17aa 30965297

[B134] Yu C. Ma X. Zhu W. Wang P. Miller K. L. Stupin J.. (2019). Scanningless and continuous 3D bioprinting of human tissues with decellularized extracellular matrix. Biomaterials 194, 1–13. 10.1016/j.biomaterials.2018.12.009 30562651 PMC6339581

[B135] Zhang C. Yuan H. Liu H. Chen X. Lu P. Zhu T.. (2015). Well-aligned chitosan-based ultrafine fibers committed teno-lineage differentiation of human induced pluripotent stem cells for Achilles tendon regeneration. Biomaterials 53, 716–730. 10.1016/j.biomaterials.2015.02.051 25890767

[B136] Zhang D. Pekkanen-Mattila M. Shahsavani M. Falk A. Teixeira A. I. Herland A. (2014). A 3D Alzheimer's disease culture model and the induction of P21-activated kinase mediated sensing in iPSC derived neurons. Biomaterials 35, 1420–1428. 10.1016/j.biomaterials.2013.11.028 24290439

[B137] Zhang Z. N. Freitas B. C. Qian H. Lux J. Acab A. Trujillo C. A.. (2016). Layered hydrogels accelerate iPSC-derived neuronal maturation and reveal migration defects caused by MeCP2 dysfunction. Proc. Natl. Acad. Sci. U.S.A. 113, 3185–3190. 10.1073/pnas.1521255113 26944080 PMC4812712

